# Cost-effectiveness of immune checkpoint inhibition and targeted treatment in combination as adjuvant treatment of patient with BRAF-mutant advanced melanoma

**DOI:** 10.1186/s12913-023-09058-7

**Published:** 2023-01-18

**Authors:** Si Ni Li, Xiaomin Wan, Liu Bao Peng, Ya Min Li, Jian He Li

**Affiliations:** 1 Clinical Nursing Teaching and Research Section, The Second Xiangya Hospital, Central South University, Changsha, 410011 China; 2 The Nethersole School of Nursing, Faculty of Medicine, The Chinese University of Hong Kong, Hongkong, China; 3 School of Health and Related Research, Faculty of Medicine, Dentistry and Health, University of Sheffield, Sheffield, UK; 4 Department of Pharmacy, The Second Xiangya Hospital, Central South University, Changsha, 410011 China; 5 Present address: The Second Xiangya Hospital, Central South University, 139 Renmin Middle Road, Changsha, 410011 Hunan China

**Keywords:** Advanced melanoma, Cost-effectiveness, Immunotherapy, Microsimulation

## Abstract

**Background:**

Immune checkpoint inhibitors (ICIs) and targeted treatments have improved the health outcomes of patients with advanced melanoma. However, due to the high cost of novel therapies, it is crucial to evaluate their value by considering both effectiveness and cost. To compare the cost-effectiveness of these novel agents (atezolizumab-vemurafenib-cobimetinib, vemurafenib-plus-cobimetinib, dabrafenib-plus-trametinib, and encorafenib-plus-binimetinib) for first-line treatment of metastatic melanoma with the BRAF^V600^ mutation.

**Methods:**

A patient-level model was developed to project the health outcomes of 4 strategies for patients with advanced melanoma. We estimated transition probabilities from the IMspire150 (ClinicalTrials.gov, NCT02908672), COMBI-AD (NCT01682083), and COLUMBUS (NCT01909453) trials using a parametric survival model. All health outcomes, including direct cost, quality-adjusted life-years (QALYs) and the incremental cost-effectiveness ratio (ICER), were estimated from the US payer perspective. Lifetime cost, QALYs, life-years (LYs), and ICERs were calculated. Univariable and probabilistic sensitivity analyses were performed to test model robustness, along with multiple scenario analyses.

**Results:**

Of the 4 competing strategies, atezolizumab-vemurafenib-cobimetinib produced the best health outcomes, and the vemurafenib-cobimetinib strategy was the least expensive option. Atezolizumab-vemurafenib-cobimetinib, dabrafenib-plus-trametinib, and vemurafenib-cobimetinib formed the cost-effective frontier, indicating that the ordered ICERs were $325,113/QALYs for dabrafenib-plus-trametinib vs. vemurafenib-cobimetinib strategies and $2,247,500/QALYs for atezolizumab-vemurafenib-cobimetinib vs. dabrafenib-plus-trametinib strategies. Encorafenib-plus-binimetinib was dominated by the other 3 competing strategies. The drug price and first-line utility significantly influenced the model utcomes.

**Conclusions:**

For BRAF-mutant advanced melanoma, the vemurafenib-cobimetinib strategy could be considered the most cost-effective treatment at the willingness-to-pay threshold of $150,000.

**Supplementary Information:**

The online version contains supplementary material available at 10.1186/s12913-023-09058-7.

## Background

Melanoma is the 5th most common cancer in the United States (US), with over 100,350 new cases diagnosed and approximately 6850 deaths occurring in 2020 [1]. The mortality risk related to melanoma is proportional to the depth of the primary tumor [2]; although approximately 98% of patients with localized early-stage melanoma survive for 5 years or more after disease diagnosis, the 5-year survival rate for advanced melanoma is only 10% in the US [3, 4]. BRAF, as the most commonly mutated gene, leads to a high mutation burden for patients with melanoma (approximately 50% of patients with metastatic melanoma have BRAF mutations) [5, 6].

While surgical excision is the main therapy for localized early-stage disease, unresectable and advanced melanoma is managed with diverse therapeutic options [7]. These novel therapies, including immune checkpoint inhibitors (ICIs) (nivolumab and atezolizumab) and targeted treatments (dabrafenib, vemurafenib, and encorafenib as BRAF inhibitors and trametinib, cobimetinib, and binimetinib as MEK inhibitors), were approved by the US Food and Drug Administration in recent years and have significantly improved the treatment landscape for metastatic melanoma [8–11].

More recent studies have reported that combinations of treatments with different mechanisms can improve efficacy, safety, and tolerability [9, 12]. In a phase III, randomized, double-blind trial (IMspire150), Gutzmer et al. evaluated the safety and efficacy of the combination of ICIs (atezolizumab) with BRAF plus MEK inhibitors (vemurafenib-plus-cobimetinib) in patients with advanced BRAF^V600^ mutation-positive melanoma [9]. The trial demonstrated that progression-free survival (PFS) was significantly prolonged in the study group (atezolizumab, vemurafenib, and cobimetinib) versus the control group (placebo, vemurafenib, and cobimetinib) (15·1 vs. 10·6 months; hazard ratio [HR] 0·78; 95% confidence interval [CI] 0·63–0·97). With the risk of adverse events (AEs) and discontinuation of treatment due to AEs being virtually similar between the study and control groups, it was concluded that the study group had no additional safety concerns to note.

Over nearly a decade, the cost of novel cancer agents has increased at unprecedented rates [13]. The cost of melanoma treatment in the US is projected to reach $3.16 billion by 2020, a 34% increase from 2010 [3]. Although previous studies have confirmed that the combination of ICIs with BRAF plus MEK inhibitors dramatically improves health outcomes in patients with BRAF^V600^ mutation-positive melanoma, it is still unknown whether considerable drug prices and intolerant AEs could be balanced by enhanced health benefits. Under the current healthcare setting, there is an increasing need to evaluate the value of different drugs in cancer in terms of both efficacy and cost to provide reasonable evidence for patients, physicians, and policy makers.

Therefore, the objective of this study was to estimate the cost-effectiveness of a combination treatment of BRAF plus MEK inhibitors with or without ICI treatments as a first-line adjuvant treatment for BRAF-mutant advanced melanoma from the US payer perspective.

## Methods

### Analytic overview

**Fig. 1:**
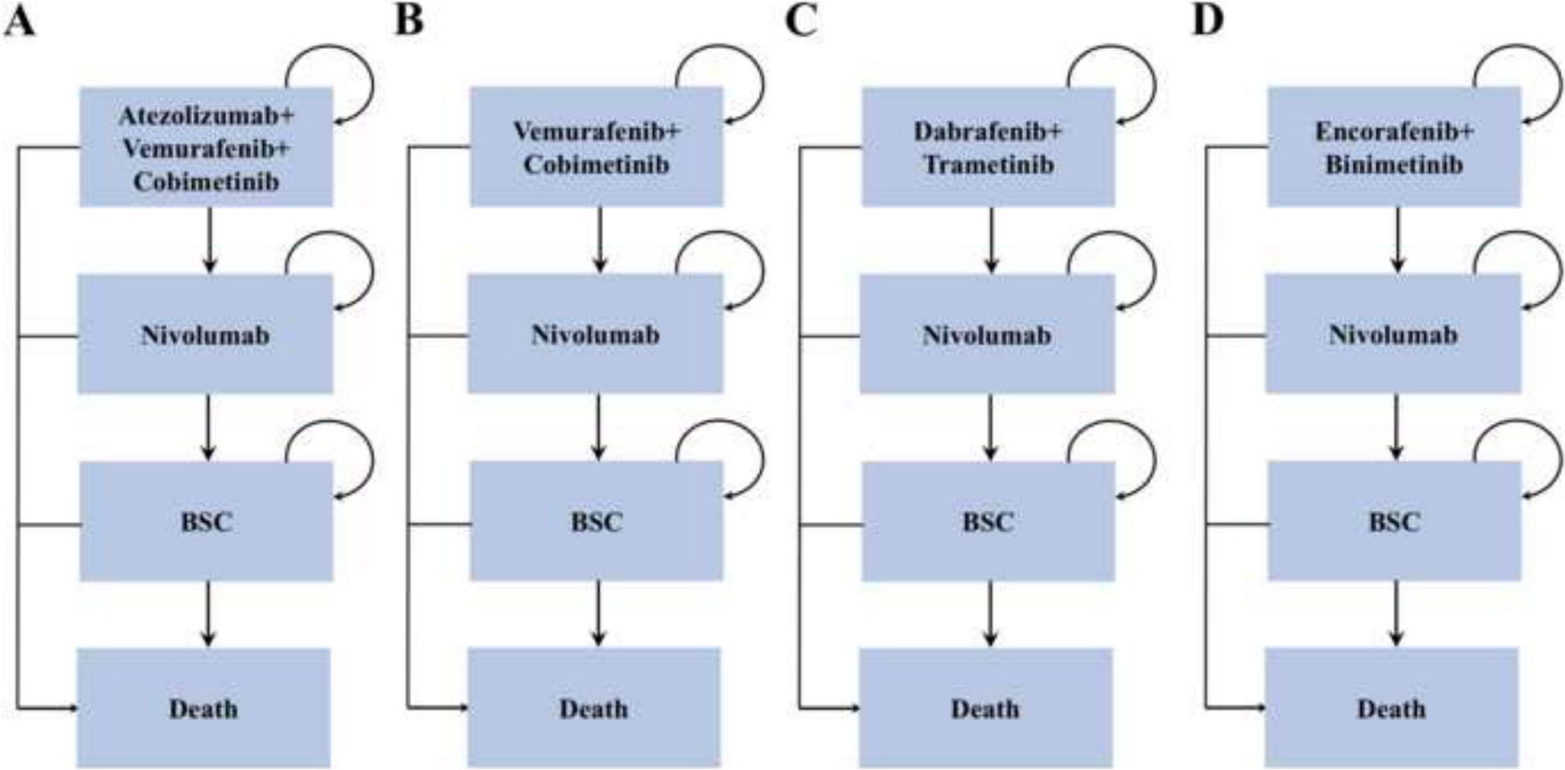
Treatment sequences. *BSC = best support care; A, B, C, and D represented 4 treatment strategies in the model

**Fig. 2:**
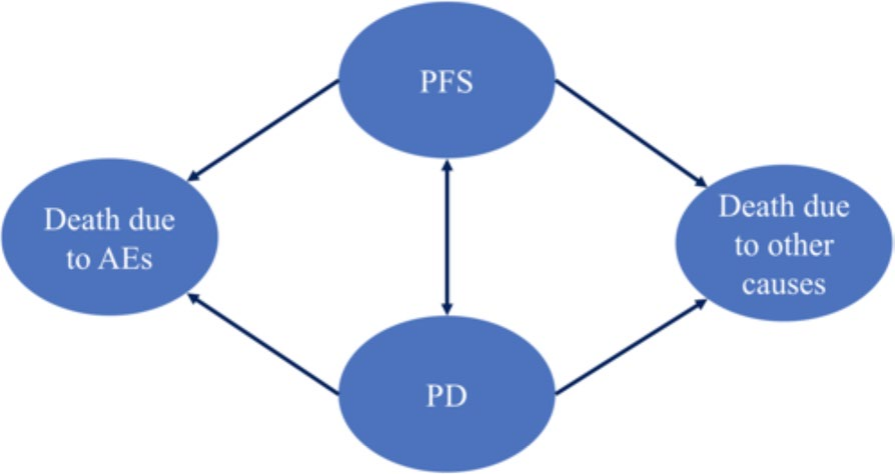
Model structure. *PFS = progression-free survival; PD = progressive disease; AEs = adverse events

A microsimulation model was constructed to estimate the lifetime cost and health outcomes of 4 treatment strategies for patients with BRAF-mutant advanced melanoma by using TreeAge 2020 Software. (Figs. 1 and 2) Each model cycle represented 28 days over a lifetime horizon. Based on the cohort enrolled in the respective randomized control trials (RCTs) published previously, we generated a 50,000 baseline sample of patients [9–11].

Patients who were included in the model were treated with 1 of 4 frontline interventions: (1) atezolizumab-vemurafenib-cobimetinib, (2) vemurafenib-plus-cobimetinib, (3) dabrafenib-plus-trametinib, or (4) encorafenib-plus-binimetinib. Patients were prescribed nivolumab as second-line treatment after disease progression, and finally, all patients were switched to the best supportive care (BSC) state until death. (Fig. 1) Dosing and administration schedules for all strategies were based on the respective RCTs and are listed in Supplementary Table 2.

The primary outcomes of the model included total cost, quality-adjusted life-years (QALYs) and life-years (LYs), which were used to estimate the incremental cost-effectiveness ratios (ICERs). We conducted this analysis from a US payer perspective, adjusting all the costs to 2021 U.S. dollars and discounting both cost and utilities by 3% annually [14]. Moreover, a willingness-to-pay threshold (WTP) of $150,000/QALY was applied in this cost-effectiveness analysis [15].

### Study population

The baseline sample in the model was constructed based on the IMspire150, COMBI-AD, and COLUMBUS trials [9–11]. Patients had a mean age of 54 years (range: 18–89 years) (calculated by averaging the mean age of the patients in the 3 RCTs), and all patients had BRAF mutation advanced melanoma. The average proportion of males is 55.5% (range: 45–60%). A total of 78.6% of patients were ranked in 0 grade using Eastern Cooperative Oncology Group (ECOG) performance status, indicating those patients were fully active and able to carry on all pre-disease performance without restriction. This study assumed that the patient characteristics of included studies were not different. The characteristics of patients were summarized in Supplementary Table 1.

### Model structure

The model was classified as four states: PFS, progress-disease (PD), discontinuation due to AEs, or death due to other causes. (Fig. 2) Patients were decided to remain on current treatment or switched to next line therapy on the basis of transition probability which was estimated by using PFS or overall survival (OS) data obtained from previous respective studies [9–11, 16, 17]. The overall mortality rate (except for BSC state) during each line of active treatment corresponded to the transition probability for death state, calculated as the background mortality rate from the 2017 US Life Table [16] and treatment-related serious AEs from RCTs [9–11, 17]. (The US Life Table is listed in Supplementary Table 3) As same as previous study, we applied Formula 1 as follows to transform the rates in the Life Table and treatment-related serious AEs to the transition probability [18]. The minimum value was taken between the probability for death state and 1 [19]. Based on the mortality data for metastatic melanoma, the probability of death from the BSC state was calculated by implementing the standard extrapolation method derived by Guyot et al. [20] Briefly, we extracted the data points from each OS Kaplan-Meier curve from the respective RCT to generate pseudoindividual patient-level data by using Getdata digital software [21]. These reconstructed survival data were then used to fit 4 standard parametric models (log-logistic, Weibull, lognormal, and exponential). On the basis of the goodness-of-fit method (Akaike information criterion), we selected the most appropriate survival distribution to estimate the transition probability.

1 $$P=1-\mathit{\exp}\left\{-r\left.t\right\}\right.$$

*The P is representing the probability; r is the rate; and t is the time.

The probabilities of patients remaining on current treatment or progressing to subsequent therapy were estimated on the basis of the PFS curves derived from respective RCTs using the same approach as the transition probabilities of OS [9–11]. The fitting results showed that lognormal distribution was the optimal survival distribution for all PFS and OS curves, except the PFS curve of vemurafenib+ cobimetinib strategy. The fitting curves of PFS and OS are presented in Supplementary Figs. 4 to 9. The transition probability of the event was calculated by using 1 to minus the ratio of the survivor function at the end of the interval to the survivor function at the beginning of the interval [18]. (Formula 2) And the survival function of lognormal and log-logistic is represented in Formula 3 and 4, respectively, so Formula 2 could be further rewritten to the probability density function of lognormal and log-logistic [18]. We also considered discontinuation of treatment related to AEs, with transition probabilities obtained from the literature [9–11]. The rate of AEs for four treatment strategies were displayed in Supplementary Table 5. All the estimated transition probabilities are displayed in Table 1. The results of survival analyses and parameter values for all fitting curves were listed in Supplementary Tables 6 and 7.

2 $$tp\left({t}_u\right)=1-S(t)/S\left(t-u\right)$$

4 $$S(t)=1-\Phi \left(\frac{\ln \left(\textrm{t}\right)-\upmu}{\sigma}\right)$$

3 $$S(t)={\left[1+\left(t/\gamma \right)\hat{\mkern6mu} \lambda \right]}^{-1}$$

**Table 1:** Input parameters of model

Parameters	Mean	Range	distribution	Reference
Survival model of PFS in the full cohort				
Atezolizumab + vemurafenib + cobimetinib	Shape = 1.375; Scale = 13.807		Loglogistic	[9]
Vemurafenib + cobimetinib	Shape = 1.663; Scale = 10.926		Loglogistic	[9]
Dabrafenib + trametinib	Shape = 1.4075; Scale = 12.9753		Loglogistic	[11]
Encorafenib + binimetinib	Shape = 1.668; Scale = 14.080		Loglogistic	[10]
Nivolumab	Shape = 1.2021; Scale = 4.6024		Loglogistic	[17]
OS in the best support care	Shape = 1.757; Scale = 7.007		Loglogistic	[21]
Probability of treatment discontinuation as a result of AE (%)				
Atezolizumab + vemurafenib + cobimetinib	13		Beta	[9]
Vemurafenib + cobimetinib	16		Beta	[9]
Dabrafenib + trametinib	26		Beta	[11]
Encorafenib + Binimetinib	13		Beta	[10]
Nivolumab	3		Beta	[17]
Probability of treatment mortality as a result of AE (%)				
Atezolizumab + vemurafenib + cobimetinib	NA		Beta	[9]
Vemurafenib + cobimetinib	NA		Beta	[9]
Dabrafenib + trametinib	1.5		Beta	[11]
Encorafenib + Binimetinib	0		Beta	[10]
Nivolumab	0		Beta	[17]
Probability of background death	–	–	–	[16]
Drug cost, $ (per mg)				
Atezolizumab	7.82	6.26–9.38	Gamma	[22]
Vemurafenib	0.16	0.13–0.19	Gamma	[23–25]
Cobimetinib	4.32	3.46–5.18	Gamma	[23–25]
Dabrafenib	0.89	0.71–1.07	Gamma	[23–25]
Trametinib	144.95	115.96–173.94	Gamma	[23–25]
Encorafenib	0.89	0.71–1.07	Gamma	[24–26]
Binimetinib	4.63	3.70–5.56	Gamma	[24, 25, 27]
Nivolumab	28.54	22.83–32.25	Gamma	[22]
Cost of best support care	4319	3455.2–5182.8	Gamma	[28]
Management of AEs, $				
Atezolizumab + vemurafenib + cobimetinib	972	687.6–1166.4	Gamma	[29]
Vemurafenib + cobimetinib	1080	864–1296	Gamma	[29]
Dabrafenib + trametinib	1229	921-1536^a^	Gamma	[28]
Encorafenib + Binimetinib	1587	1269.6–1904.4	Gamma	[29]
Nivolumab	2688	2150.4–3225.6	Gamma	[25]
Administration cost				
IV infusion, single or initial drug (≤1 hour)	148.3	118.64–177.93	Gamma	[30]
Utilities				
Complete/partial response	0.88	0.70–1.00	Beta	[4]
Stable disease	0.80	0.64–0.96	Beta	[4]
Progressive disease	0.52	0.42–0.62	Beta	[4]
Disutility due to AEs (grade ≥ 3)	0.077	0.074–0.08^a^	Beta	[28]
Average patient weight (kg)	70		Normal	[4]
Terminal care, $	17,346	13,009 – 21682^a^	Gamma	[28]

^a^The range is the reported or estimated 95% CI

*the Φ is representing the cumulative distribution function of the standard normal distribution, the μ is representing the mean, σ is the standard deviation, γ is the shape of the distribution, and the λ is the scale.

### Costs and utilities

All the model input parameters, including the costs and utilities, are listed in Table 1. In this model, we incorporated only direct costs, including drugs, administration, BSC, management of AEs, and terminal care. The unit costs of atezolizumab and nivolumab were derived from the 2021 average sale price from the Centers for Medicare & Medicaid Services (CMS) [22]. The prices of oral drugs, including vemurafenib, cobimetinib, dabrafenib, trametinib, encorafenib, and binimetinib, were based on public databases and the literature [23–27]. The 2021 average wholesale price from RED BOOK Online was discounted at a rate of 17% to account for contract pricing and to be consistent with evaluates for Medicare reimbursement [24]. Although the average patient weight is 74.7 kg in the US, a patient weight of 70 kg was used to calculate the total cost of treatments so that the weight loss effects of the advanced disease could be taken into consideration [4]. The administration fee was derived from the 2021 CMS Physician Fee Schedule [30]. The overall costs related to BSC, management of grade 3 or 4 AEs and terminal care were obtained from the published literature [25, 28, 29].

The mean utility for each health state was obtained from published analyses. We allocated a utility of 0.88 for patients who had a complete or partial response to therapy, 0.80 for patients in first-line treatment, and 0.52 for progressive disease [31, 32]. The utility decrement (− 0.077) was also adopted to specify the reduction in the valued QALY for an adverse drug reaction [4]. QALYs are a way to measure individual- or group-obtained health benefits (length of life), which are adjusted to reflect the quality of life. In this study, QALYs were estimated by calculating the years of life remaining for the patient with BRAF-mutant advanced melanoma following our predefined treatment sequences and weighing over the lifetime horizon with a quality-of-life score. In addition, 1 QALY is equal to 1 year of life in full health. The calculation of the LYs was the same as that of the QALYs; however, LYs was regarded as the health state of a patient in perfect health (the utility was equal to 1).

### Sensitivity analyses

Multiple sensitivity analyses were performed to test the uncertainty of the model and to evaluate the robustness of our outcomes. In the univariable sensitivity analyses, we varied the model parameters based on their upper and lower limitations by using their 95% CIs or changing them by 20% from baseline to examine the impact of variables on outcomes, in accordance with the existing approach [4, 33, 34]. Moreover, a Monte Carlo simulation of 3000 iterations of 6000 patients was generated to perform probability sensitivity analyses (PSAs) by using suitable distributions to sample the key model parameters. Utilities were represented by beta distribution, costs by gamma, and weight and median starting age by normal distribution. Based on PSA, a cost-effectiveness acceptability curve (CEAC) was developed to portray the likelihood that a competing strategy would be considered a cost-effective option at different WTP thresholds for health benefits (QALYs).

We also incorporated 3 scenario analyses in this study. First, to evaluate the influence of survival curve extrapolations simulated in the model, the time horizon was varied in different time spans (10, 20, and 30 years). Second, we assumed that some patients would experience discontinuation and switch to the BSC state in a certain proportion (10–30%). Finally, the price of atezolizumab was decreased to 75, 50%, or 25% of its original cost.

## Results

### Base-case analysis

In our examination of 4 therapy strategies incrementally (Table 2), the vemurafenib-plus-cobimetinib, dabrafenib-plus-trametinib, and atezolizumab-vemurafenib-cobimetinib strategies constituted the cost-effective frontier (Supplementary Fig. 1). Supplementary Fig. 1 depicted that the vemurafenib-plus-cobimetinib strategy had the lowest cost, and compared with this strategy, the dabrafenib-plus-trametinib strategy gained an incremental 0.32 QALYs with an additional cost of $102,116, which led to an ICER of $325,113/QALY and dominated the encorafenib-plus-binimetinib strategy. The encorafenib-plus-binimetinib strategy was also dominated by the atezolizumab-vemurafenib-cobimetinib strategy, which produced the greatest health outcomes with an extra 0.10 QALYs and $227,808 compared with the dabrafenib-plus-trametinib strategy. The ICER of the atezolizumab-vemurafenib-cobimetinib vs dabrafenib-plus-trametinib strategy over dabrafenib-plus-trametinib vs vemurafenib-plus-cobimetinib strategy was $1,922,387/QALY.

**Table 2:** Base-Case results

Strategy	Total cost	LY	QALY	ICER^a^
Vemurafenib + cobimetinib	353,457	2.34	1.96	Dominate
Encorafenib + binimetinib	588,988	2.64	2.22	Dominated
Dabrafenib + trametinib	455,573	2.72	2.28	325,113
Atezolizumab + vemurafenib + cobimetinib	683,381	2.83	2.38	2,247,500

*LY* Life year; *QALY* Quality-adjusted life year, *ICER* Incremental cost-effectiveness ratio

^a^The ICER was compared with the next-best nondominated option

### Univariable sensitivity and probability analyses

**Fig. 3:**
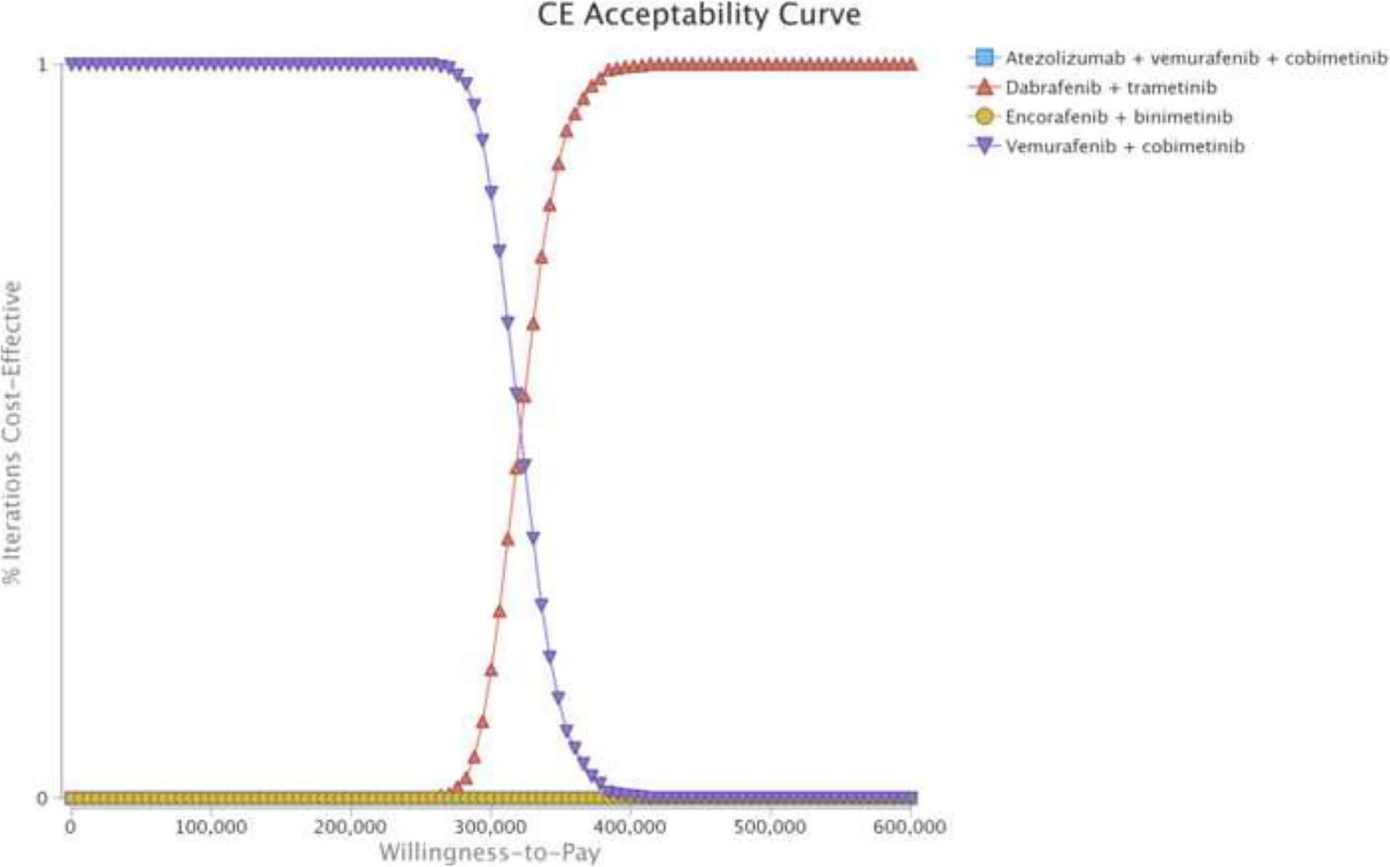
Acceptability Curves Comparing the Cost-Effectiveness of 4 Competing Strategies

In the comparison between the dabrafenib-plus-trametinib and vemurafenib-plus-cobimetinib strategies, the univariable sensitivity analyses showed that the model outcome was significantly influenced by the cost of dabrafenib, utility of first-line treatment, and the cost of vemurafenib (Supplementary Fig. 2). In the comparison between the atezolizumab-vemurafenib-cobimetinib and vemurafenib-plus-cobimetinib strategies, the utility of first-line treatment, price of atezolizumab, and discount rate were the key drivers for the model outcomes (Supplementary Fig. 3). Other model parameters, such as the decrement utility and the cost of AE management, had a mild impact on our estimated ICER. The CEAC illustrated that vemurafenib-plus-cobimetinib has a 100% probability of being considered a cost-effective option at a WTP threshold of $150,000/QALY when compared with the other 3 competing strategies (Fig. 3).

### Scenario analyses

The first scenario analyses determined that although most of the total costs (95%) were produced in the first 5 years of the time horizon, which resulted in the ICERs of the first 5 years being higher than the base-case results, patients continued to obtain health benefits after 5 years. The ICERs changed slightly when we varied the time horizon to 10 and 20 years, and with increasing treatment duration, the ICERs decreased. A small percentage of patients switched to the BSC phase after disease progression from first- or second-line therapy rather than receiving next-line treatment. In the second scenario, analyses showed that adjusting for the small proportion of patients switched to the BSC state did not dramatically change our model conclusions, with ICERs for the atezolizumab-vemurafenib-cobimetinib versus vemurafenib-plus-cobimetinib strategies of $745,702/QALY and $720,376/QALY when simulating 30 and 10% of individuals transitioning to the BSC phase, respectively. In our final scenario analyses, we demonstrated that although decrements in the costs for atezolizumab used in the first-line therapy of 25, 50 and 75% would result in lower ICERs of 266,193/QALY, $443,793/QALY, and 611,481/QALY, respectively, it is still unlikely for atezolizumab-vemurafenib-cobimetinib versus vemurafenib-plus-cobimetinib strategies to be considered a cost-effectiveness option. All the scenario analysis outcomes are listed in Supplementary Table 4.

## Discussion

After the results of several respective RCTs, the Food and Drug Administration (FDA) approved combination vemurafenib-plus-cobimetinib, dabrafenib-plus-trametinib, and encorafenib-plus-binimetinib for patients with melanoma, and this adjuvant treatment became a new standard of care for patients with BRAF-mutant melanoma. Compared with 20 years ago, the annual total medical expenditure of melanoma increased from $249 million to over 600 million, which might be associated with a high cost of diagnosis, drug acquisition, treatment of metastases, AE management and end-of-life care [2, 35–38]. Unlike the regulatory body of the United Kingdom (the National Institution for Health and Care Excellence), the FDA did not take cost-effectiveness into consideration when making decisions concerning drug approval [39]. Therefore, CMS also does not take into account cost-effectiveness when making decisions regarding reimbursement and coverage [39]. Although immunotherapy dramatically enhances health outcomes in patients with BRAF-mutant melanoma, the optimal combination of immune-based treatments is still unknown from a value standpoint.

This study reveals that among the 4 competing arms, the upfront use of atezolizumab-vemurafenib-cobimetinib maximized health outcomes, followed by the dabrafenib-plus-trametinib and vemurafenib-cobimetinib strategies. Although these 3 strategies constituted the frontier line, they could not be regarded as the cost-effectiveness option because the ICER significantly exceeded the US WTP threshold.

To our knowledge, this is the first cost-effectiveness analysis incorporating different adjuvant treatment strategies for patients with BRAF-mutant melanoma. In addition, this study has multiple important strengths. First, this study was conducted on the basis of a series of randomized, double-blind phase III clinical trials. Second, we predefined appropriate frontline treatment to reflect the current standard of care and most advances in the treatment of BRAF-mutant melanoma. Third, we considered the AEs in the model, incorporating not only treatment discontinuation or death because of AEs but also the costs and decrement utility associated with medical toxicity. Fourth, the microsimulation model applied in this study could effectively reflect the heterogeneity in patients’ baseline characteristics and depict specific clinical pathways, as well as incorporate the influence of history on future events [40, 41]. Finally, our assumption helps this study represent clinical practice more appropriately. For example, we assumed that a certain proportion of patients will switch to the BSC state instead of receiving next-line treatment.

This study also had some limitations that should be considered. First, we developed an indirect comparison between each strategy by obtaining data from multiple respective RCTs due to the paucity of head-to-head data. This leads to a weakness due to the comparison is predicated on the assumption that there are no significant differences in patient characteristics amongst all included studies. Although this assumption would bring some biases to our model, this method was widely applied in economic analysis studies [42–44], and we validated the survival curves we fitted in our model (Supplementary Figs. 4 to 9). Therefore, we appeal for a more direct comparison study in the future, and if the data of real-world data or long-term RCTs are available, we will update our conclusion. Second, our study did not incorporate adjuvant treatment (BRAF inhibitor + MEK inhibitor) as a second-line therapy because of a lack of data. Of the included RCTs, only the COLUMBUS trial had a small proportion of patients who had received previous treatment. As the RCT was conducted in the near future for patients with BRAF-mutant melanoma, future economic analyses should take adjuvant treatment as second-line treatment into account in the decision-making process. Finally, although the health utilities we used in the model were derived from a previously published cost-effectiveness study for advanced melanoma, it may not precisely reflect the patients we simulated in the model. Accuracy and robustness might be enhanced when utilities calculated for patients with BRAF-mutant melanoma in patients with immunotherapy are available in the future.

## Conclusion

From the US payer perspective, the vemurafenib-plus-cobimetinib strategy could be regarded as the most cost-effective treatment for patients with BRAF-mutant advanced melanoma when we set the WTP threshold at 150,000/QALY compared with the other 3 competing strategies.

## Supplementary Information

**Additional file 1: Supplementary Table 1.** Summary table of included studies. **Supplementary Table 2.** Drug dose and costs. **Supplementary Table 3.** Background mortality rate. **Supplementary Table 4.** The results of scenario analyses. **Supplementary Table 5.** The Rate of Adverse Events for Four Treatment Strategies. **Supplementary Table 6.** The results of survival analyses for all fitting curves. **Supplementary Table 7.** The parameter values of survival analyses for all fitting curves. **Supplementary Fig. 1.** The Cost-Effective Frontier of 4 Different Competing Strategies. **Supplementary Fig. 2.** Tornado Diagrams Showing the Effect of Lower and Upper Values of Each Parameter on the ICERs of the Pembrolizumab-plus-Axitinib Versus Sunitinib Strategy. **Supplementary Fig. 3.** Tornado Diagrams Showing the Effect of Lower and Upper Values of Each Parameter on the ICERs of the Atezolizumab-Vemurafenib-Cobimetinib Versus Vemurafenib-plus-Cobimetinib Strategies. **Supplementary Fig. 4.** Parametric Distributions for First-Line Treatment (Atezolizumab + vemurafenib + cobimetinib strategy). **Supplementary Fig. 5.** Parametric Distributions for First-Line Treatment (Vemurafenib + cobimetinib strategy). **Supplementary Fig. 6.** Parametric Distributions for First-Line Treatment (Dabrafenib + trametinib strategy). **Supplementary Fig. 7.** Parametric Distributions for First-Line Treatment (Encorafenib + binimetinib strategy). **Supplementary Fig. 8.** Parametric Distributions for Second-Line Treatment. **Supplementary Fig. 9.** Parametric Distributions for Best Support Care State. The ISPOR CHEERs checklist.

## Data Availability

All data generated or analyzed during this study are included in this published article as Supplementary information files.

## Abbreviations

AEs: Adverse events
BSC: Best supportive care
CEAC: Cost-effectiveness acceptability curve
CI: Confidence interval
CMS: Centers for Medicare & Medicaid Services
ECOG: Eastern Cooperative Oncology Group
FDA: Food and Drug Administration
HR: Hazard ratio
ICER: Incremental cost-effectiveness ratio
ICIs: Immune checkpoint inhibitors
LYs: Life-years
OS: Overall survival
PD: Progress-disease
PFS: Progression-free survival
PSAs: Probability sensitivity analyses
QALYs: Quality-adjusted life-years
RCTs: Randomized control trials
US: United States
WTP: Willingness-to-pay threshold
