# Supplementary material for: Cost-effectiveness of immune checkpoint inhibition and targeted treatment in combination as adjuvant treatment of patient with BRAF-mutant advanced melanoma
Source: BMC Health Serv Res. 2023 Jan 18;23:49. doi: 10.1186/s12913-023-09058-7 (PMC9847087; doi:10.1186/s12913-023-09058-7)
Supplement: Supplementary file 1 — Additional file 1: Supplementary Table 1. Summary table of included studies. Supplementary Table 2. Drug dose and costs. Supplementary Table 3. Background mortality rate. Supplementary Table 4. The results of scenario analyses. Supplementary Table 5. The Rate of Adverse Events for Four Treatment Strategies. Supplementary Table 6. The results of survival analyses for all fitting curves. Supplementary Table 7. The parameter values of survival analyses for all fitting curves. Supplementary Fig. 1. The Cost-Effective Frontier of 4 Different Competing Strategies. Supplementary Fig. 2. Tornado Diagrams Showing the Effect of Lower and Upper Values of Each Parameter on the ICERs of the Pembrolizumab-plus-Axitinib Versus Sunitinib Strategy. Supplementary Fig. 3. Tornado Diagrams Showing the Effect of Lower and Upper Values of Each Parameter on the ICERs of the Atezolizumab-Vemurafenib-Cobimetinib Versus Vemurafenib-plus-Cobimetinib Strategies. Supplementary Fig. 4. Parametric Distributions for First-Line Treatment (Atezolizumab + vemurafenib + cobimetinib strategy). Supplementary Fig. 5. Parametric Distributions for First-Line Treatment (Vemurafenib + cobimetinib strategy). Supplementary Fig. 6. Parametric Distributions for First-Line Treatment (Dabrafenib + trametinib strategy). Supplementary Fig. 7. Parametric Distributions for First-Line Treatment (Encorafenib + binimetinib strategy). Supplementary Fig. 8. Parametric Distributions for Second-Line Treatment. Supplementary Fig. 9. Parametric Distributions for Best Support Care State. The ISPOR CHEERs checklist. [file 12913_2023_9058_MOESM1_ESM.docx]

**Appendix**

Supplementary table 1. Summary table of included studies

Supplementary table 2. Drug dose and costs

Supplementary table 3. Background mortality rate

Supplementary table 4. The results of scenario analyses

Supplementary table 5. The Rate of Adverse Events for Four Treatment Strategies.

Supplementary table 6. The results of survival analyses for all fitting curves

Supplementary table 7. The parameter values of survival analyses for all fitting curves

Supplementary figure 1. The Cost-Effective Frontier of 4 Different Competing Strategies

Supplementary figure 2. Tornado Diagrams Showing the Effect of Lower and Upper Values of Each Parameter on the ICERs of the Pembrolizumab-plus-Axitinib Versus Sunitinib Strategy

Supplementary figure 3. Tornado Diagrams Showing the Effect of Lower and Upper Values of Each Parameter on the ICERs of the Atezolizumab-Vemurafenib-Cobimetinib Versus Vemurafenib-plus-Cobimetinib Strategies

Supplementary figure 4: Parametric Distributions for First-Line Treatment (Atezolizumab + vemurafenib + cobimetinib strategy)

Supplementary figure 5: Parametric Distributions for First-Line Treatment (Vemurafenib + cobimetinib strategy)

Supplementary figure 6: Parametric Distributions for First-Line Treatment (Dabrafenib + trametinib strategy)

Supplementary figure 7: Parametric Distributions for First-Line Treatment (Encorafenib + binimetinib strategy)

Supplementary figure 8: Parametric Distributions for Second-Line Treatment

Supplementary figure 9: Parametric Distributions for Best Support Care State

The ISPOR CHEERs checklist

**Supplementary table 1. Summary table of included studies.**

|  | Key characteristic | | | | | | | | | | | |
| --- | --- | --- | --- | --- | --- | --- | --- | --- | --- | --- | --- | --- |
| Study | Study design | Treatment | Sample size | Age (yrs) | Male (%) | ECOG performance status | | | | BRAF mutation status (%) | | |
|  |  |  |  |  |  | 0 | 1 | | Unknow | BRAF^V600E^ | BRAF^V600K^ | |
| First-line treatment | | | | | | | | | | | | |
| IMspire150 | Phase III double-blind, placebo-controlled RCT | ATE-VEM-COB  VEM-COB | 256  258 | 54  53.5 | 59  58 | 76  77 | 24  22 | 0  2 | | 77  71 | | 11  11 |
| COMBI-AD | Phase III double-blind, placebo-controlled RCT | DAB-TRA | 438 | 50 | 45 | 92 | 8 | 1 | | 91 | | 9 |
| COLUMBUS | Phase III open-label RCT | ENC-BIN | 192 | 57 | 60 | 71 | 29 | - | | 89 | | 11 |
| Second-line treatment | | | | | | | | | | | | |
| CheckMate 037 | Phase III open-label RCT | Nivolumab | 272 | 59 | 65 | 60 | 40 | - | | 22 | | |

***** ATE-VEM-COB = atezolizumab-vemurafenib-cobimetinib; VEM-COB = vemurafenib-plus-cobimetinib; DAB-TRA = dabrafenib-plus-trametinib; ENC-BIN = encorafenib-plus-binimetinib.

**Supplementary table 2. Drug dose and costs**

| Drug | Dose | Route | Unit Price ($) | Cost for 1 model cycle ($, 28 days) |
| --- | --- | --- | --- | --- |
| Atezolizumab + vemurafenib + cobimetinib strategy | | | | |
| Cycle 1 | | | | |
| Atezolizumab | - | - | - | - |
| Vemurafenib | 960 mg twice-daily (for 21 day) followed by 720 mg vemurafenib twice-daily | Oral | 0.16 | 8064 |
| Cobimetinib | 60 mg once-daily | Oral | 4.32 | 7257.6 |
| From cycle 2 onwards | | | | |
| Atezolizumab | 840 mg on day 1 and 15 | IV | 7.82 | 13137.6 |
| Vemurafenib | 720 mg twice daily | Oral | 0.16 | 6451.2 |
| Cobimetinib | 60 mg once-daily (21 days on–7 days off) | Oral | 4.32 | 5443.2 |
| vemurafenib + cobimetinib strategy | | | | |
| Cycle 1 | | | | |
| Vemurafenib | 960 mg twice-daily (for 21 day) followed by vemurafenib 960 mg for 7 days | Oral | 0.16 | 7526.4 |
| Cobimetinib | 60 mg once-daily | Oral | 4.32 | 7257.6 |
| From cycle 2 onwards | | | | |
| Vemurafenib | 960 mg vemurafenib twice daily | Oral | 0.16 | 8601.6 |
| Cobimetinib | 60 mg cobimetinib once-daily (21 days on–7 days off) | Oral | 4.32 | 5443.2 |
| Dabrafenib + trametinib strategy | | | | |
| Dabrafenib | 150 mg twice daily for 12 months | Oral | 0.89 | 7476 |
| Trametinib | 2 mg once daily for 12 months | Oral | 144.95 | 8117.2 |
| Encorafenib + Binimetinib strategy | | | | |
| Encorafenib | 450 mg once daily | Oral | 0.89 | 11214 |
| Binimetinib | 45 mg twice daily | Oral | 4.63 | 11667.6 |
| Second-line treatment | | | | |
| Nivolumab | 3 mg/kg every 2 weeks | IV | 28.54 | 11986.8 |

**Supplementary table 3. Background mortality rate**

Estimates of background mortality rate for each age are provided in the US life table; Arias E, Heron M, Xu J. United States Life Tables, 2019. Natl Vital Stat Rep. 2019; 68:1-65.

| Age (years) | Background | Age (years) | Background | Age (years) | Background |
| --- | --- | --- | --- | --- | --- |
| 18 | 0.000603 | 54 | 0.007003 | 90 | 0.166829 |
| 19 | 0.000698 | 55 | 0.007607 | 91 | 0.185047 |
| 20 | 0.000795 | 56 | 0.008219 | 92 | 0.204441 |
| 21 | 0.000889 | 57 | 0.008857 | 93 | 0.224919 |
| 22 | 0.000970 | 58 | 0.009542 | 94 | 0.246354 |
| 23 | 0.001424 | 59 | 0.010285 | 95 | 0.26890 |
| 24 | 0.001497 | 60 | 0.011098 | 96 | 0.291442 |
| 25 | 0.001561 | 61 | 0.011952 | 97 | 0.314700 |
| 26 | 0.001624 | 62 | 0.012814 | 98 | 0.338142 |
| 27 | 0.001682 | 63 | 0.013657 | 99 | 0.361537 |
| 28 | 0.001737 | 64 | 0.014502 | 100 | 1 |
| 29 | 0.001792 | 65 | 0.015384 |  |  |
| 30 | 0.001847 | 66 | 0.016444 |  |  |
| 31 | 0.001900 | 67 | 0.017624 |  |  |
| 32 | 0.001952 | 68 | 0.018968 |  |  |
| 33 | 0.002003 | 69 | 0.029586 |  |  |
| 34 | 0.002053 | 70 | 0.022109 |  |  |
| 35 | 0.002111 | 71 | 0.024359 |  |  |
| 36 | 0.002174 | 72 | 0.026347 |  |  |
| 37 | 0.002233 | 73 | 0.028810 |  |  |
| 38 | 0.002285 | 74 | 0.031309 |  |  |
| 39 | 0.002340 | 75 | 0.034486 |  |  |
| 40 | 0.002413 | 76 | 0.038026 |  |  |
| 41 | 0.002516 | 77 | 0.042286 |  |  |
| 42 | 0.002649 | 78 | 0.046547 |  |  |
| 43 | 0.002811 | 79 | 0.051534 |  |  |
| 44 | 0.002999 | 80 | 0.057008 |  |  |
| 45 | 0.003203 | 81 | 0.062923 |  |  |
| 46 | 0.003433 | 82 | 0.069911 |  |  |
| 47 | 0.003709 | 83 | 0.078099 |  |  |
| 48 | 0.004047 | 84 | 0.086754 |  |  |
| 49 | 0.004445 | 85 | 0.096549 |  |  |
| 50 | 0.004874 | 86 | 0.106472 |  |  |
| 51 | 0.005331 | 87 | 0.119677 |  |  |
| 52 | 0.005844 | 88 | 0.134128 |  |  |
| 53 | 0.006408 | 89 | 0.149846 |  |  |

**Supplementary table 4. The results of scenario analyses**.

| Strategy | Total cost | LY | QALY | ICER |
| --- | --- | --- | --- | --- |
| Scenario 1 | | | | |
| 5 years | | | | |
| Vemurafenib + cobimetinib | 312875 | 1.99 | 1.65 | Dominate |
| Encorafenib + binimetinib | 517534 | 2.20 | 1.84 | Extend dominated |
| Dabrafenib + trametinib | 383970 | 2.18 | 1.82 | 402359 |
| Atezolizumab + vemurafenib + cobimetinib | 577914 | 2.23 | 1.89 | 3201604 |
| 10 years | | | | |
| Vemurafenib + cobimetinib | 347548 | 2.24 | 1.88 | Dominate |
| Encorafenib + binimetinib | 578454 | 2.50 | 2.14 | Dominated |
| Dabrafenib + trametinib | 447242 | 2.56 | 2.18 | 335118 |
| Atezolizumab + vemurafenib + cobimetinib | 670036 | 2.66 | 2.26 | 2829557 |
| 20 years | | | | |
| Vemurafenib + cobimetinib | 357053 | 2.33 | 1.99 | Dominate |
| Encorafenib + binimetinib | 596896 | 2.60 | 2.25 | Dominated |
| Dabrafenib + trametinib | 458757 | 2.66 | 2.30 | 325391 |
| Atezolizumab + vemurafenib + cobimetinib | 683692 | 2.78 | 2.40 | 2340589 |
| Scenario 2 | | | | |
| 10% patents switch to BSC | | | | |
| Vemurafenib + cobimetinib | 339320 | 2.27 | 1.89 | Domimate |
| Encorafenib + binimetinib | 578851 | 2.56 | 2.16 | Dominated |
| Dabrafenib + trametinib | 443650 | 2.62 | 2.21 | 325092 |
| Atezolizumab + vemurafenib + cobimetinib | 670693 | 2.73 | 0.10 | 2225006 |
| 30% patents switch to BSC | | | | |
| Vemurafenib + cobimetinib | 317539 | 2.10 | 1.77 | Dominate |
| Encorafenib + binimetinib | 558446 | 2.41 | 2.06 | Dominated |
| Dabrafenib + trametinib | 424906 | 2.48 | 2.12 | 303800 |
| Atezolizumab + vemurafenib + cobimetinib | 653105 | 2.61 | 2.22 | 2380948 |
| Scenario 3 | | | | |
| Adjust nivolumab 75% of its original price in the first-line setting. | | | | |
| Vemurafenib + cobimetinib | 357509 | 2.34 | 1.98 | - |
| Atezolizumab + vemurafenib + cobimetinib | 620446 | 2.82 | 2.41 | 611481 |
| Adjust nivolumab 50% of its original price in the first-line setting. | | | | |
| Vemurafenib + cobimetinib | 353758 | 2.34 | 1.97 | - |
| Atezolizumab + vemurafenib + cobimetinib | 544589 | 2.82 | 2.40 | 443793 |
| Adjust nivolumab 25% of its original price in the first-line setting. | | | | |
| Vemurafenib + cobimetinib | 358197 | 2.35 | 1.99 | - |
| Atezolizumab + vemurafenib + cobimetinib | 475322 | 2.80 | 2.43 | 266193 |

**Supplementary table 5. The Rate of Adverse Events for Four Treatment Strategies.**

| Arms | The probability of AE caused discontinuation | The probability of AE caused death | References |
| --- | --- | --- | --- |
| Atezolizumab +vemurafenib + cobimetinib | 0.13 | 0 | 1 |
| Vemurafenib + cobimetinib | 0.16 | 0 | 1 |
| Dabrafenib +trametinib | 0.26 | 0.015 | 2 |
| Encorafenib +binimetinib | 0.13 | 0 | 3 |
| Nivolumab | 0.03 | 0 | 4 |

References:

1. Gutzmer R, Stroyakovskiy D, Gogas H, et al. Atezolizumab, vemurafenib, and cobimetinib as first-line treatment for unresectable advanced BRAF(V600) mutation-positive melanoma (IMspire150): primary analysis of the randomised, double-blind, placebo-controlled, phase 3 trial. Lancet (London, England). 2020;395(10240):1835-1844.

2. Long GV, Hauschild A, Santinami M, et al. Adjuvant Dabrafenib plus Trametinib in Stage III BRAF-Mutated Melanoma. N Engl J Med. 2017;377(19):1813-1823.

3. Dummer R, Ascierto PA, Gogas HJ, et al. Overall survival in patients with BRAF-mutant melanoma receiving encorafenib plus binimetinib versus vemurafenib or encorafenib (COLUMBUS): a multicentre, open-label, randomised, phase 3 trial. Lancet Oncol. 2018;19(10):1315-1327.

4. Weber JS, D'Angelo SP, Minor D, et al. Nivolumab versus chemotherapy in patients with advanced melanoma who progressed after anti-CTLA-4 treatment (CheckMate 037): a randomised, controlled, open-label, phase 3 trial. Lancet Oncol. 2015;16(4):375-384.

**Supplementary table 6. The results of survival analyses for all fitting curves.**

| Distribution | Progressive-free survival | | | | | OS |
| --- | --- | --- | --- | --- | --- | --- |
|  | First-line | | | | Second-line |  |
|  | Atezolizumab+ vemurafenib+ cobimetinib | vemurafenib+ cobimetinib | Dabrafenib+ trametinib | Encorafenib+ binimetinib | Nivolumab | BSC |
| Exponential | 905.99 | 1087.11 | 2423.072 | 531.04 | 839.95 | 492.92 |
| Weibull | 906.46 | 1080.49 | 2413.763 | 524.49 | 821.53 | 493.23 |
| Log-logistic | 898.07 | **1067.13** | 2341.131 | 519.16 | 782.09 | 477.09 |
| Lognormal | **895.47** | 1076.68 | **2333.435** | **516.14** | **778.96** | **476.49** |

PFS: Progressive-free survival; OS: Overall survival; BSC: Best support care.

**Supplementary table 7. The parameter values of survival analyses for all fitting curves.**

|  | Exponential (SE) | Weibull (SE) | Lognormal (SE) | Log-logistic (SE) |
| --- | --- | --- | --- | --- |
| Atezolizumab+ vemurafenib+ cobimetinib | Rate = 0.0463 (0.0044) | Shape = 1.1028 (0.0853)  Scale = 20.6978 (1.8859) | Mean = 2.6463 (0.1053)  Sd = 1.2683 (0.0886) | Shape = 1.375 (0.105)  Scale = 13.807 (1.363) |
| vemurafenib+ cobimetinib | Rate = 0.0596 (0.005) | Shape = 1.2275 (0.0822)  Scale = 16.3221 (1.1191) | Mean = 2.4028 (0.0824)  Sd = 1.0941 (0.0658) | Shape = 1.663 (0.113)  Scale = 10.926 (0.830) |
| Dabrafenib+ trametinib | Rate = 0.03659 (0.00218) | Shape = 0.8663 (0.0381)  Scale = 27.0674 (1.8639) | Mean = 2.6865 (0.0711)  Sd = 1.3105 (0.0553) | Shape = 1.307 (0.061)  Scale = 13.677 (0.979) |
| Encorafenib+ binimetinib | Rate = 0.04358 (0.00545) | Shape = 1.370 (0.139)  Scale = 19.271 (1.934) | Mean = 2.6668 (0.1142)  Sd = 1.0439 (0.0967) | Shape = 1.668 (0.167)  Scale = 14.080 (1.497) |
| Nivolumab | Rate = 0.09015 (0.00813) | Shape = 0.7683 (0.0477)  Scale = 10.8711 (1.2778) | Mean = 1.6439 (0.1178)  Sd = 1.4534 (0.0881) | Shape = 1.2021 (0.0829)  Scale = 4.6024 (0.5468) |
| BSC | Rate = 0.0857 (0.0102) | Shape = 1.1161 (0.0917)  Scale = 11.7287 (1.2504) | Mean = 1.9695 (0.1092)  Sd = 0.9994 (0.0816) | Shape = 1.757 (0.164)  Scale = 7.007 (0.753) |

Supplementary figure 1. The Cost-Effective Frontier of 4 Different Competing Strategies.


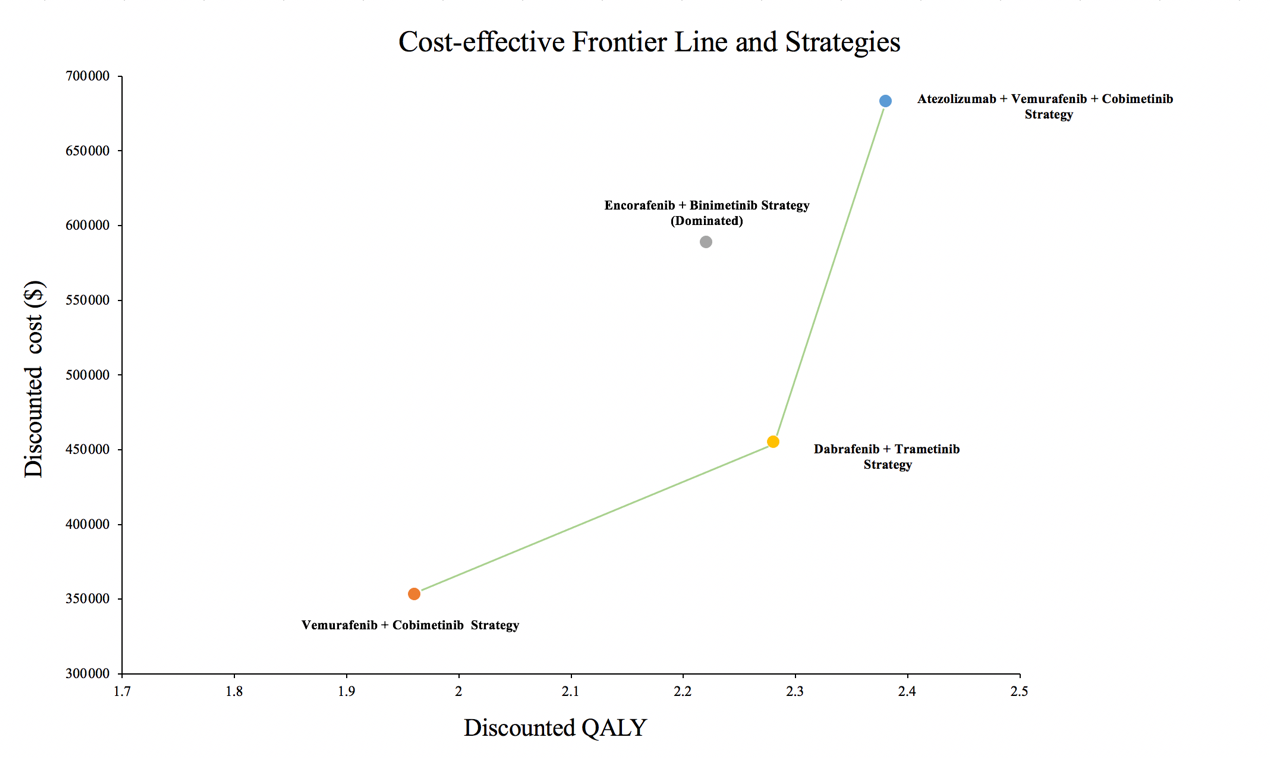


Supplementary figure 2. Tornado Diagrams Showing the Effect of Lower and Upper Values of Each Parameter on the ICERs of the Dabrafenib-plus-Trametinib and Vemurafenib-plus-Cobimetinib Strategies.


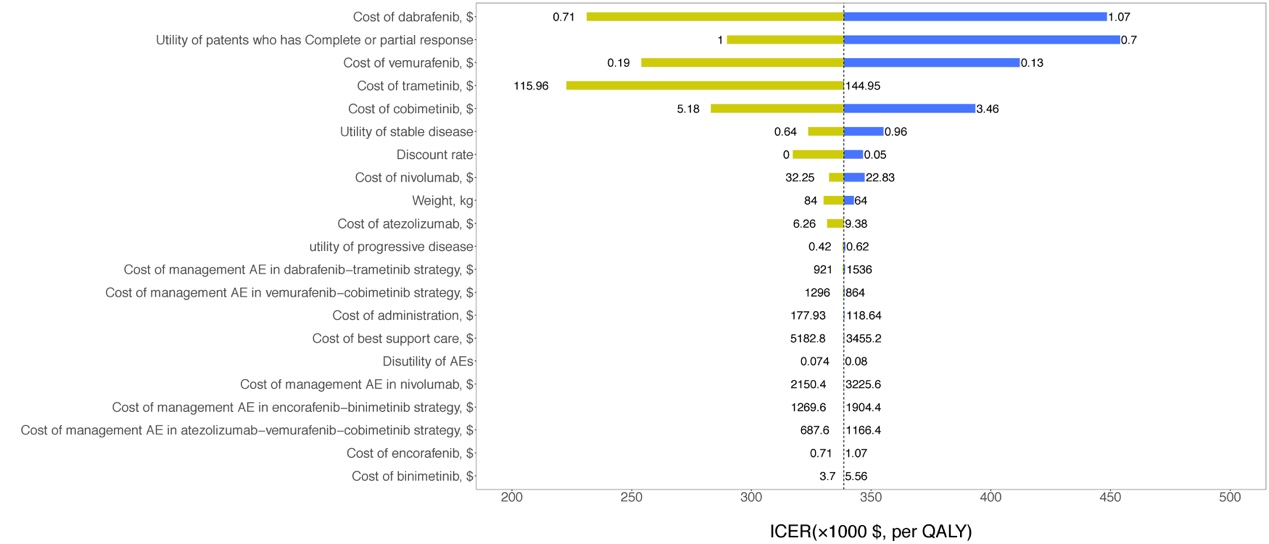


Supplementary figure 3. Tornado Diagrams Showing the Effect of Lower and Upper Values of Each Parameter on the ICERs of the Atezolizumab-Vemurafenib-Cobimetinib Versus Vemurafenib-plus-Cobimetinib Strategies

Supplementary figure 4: Parametric Distributions for First-Line Treatment (Atezolizumab + vemurafenib + cobimetinib strategy). KM = Kaplan-Meier.


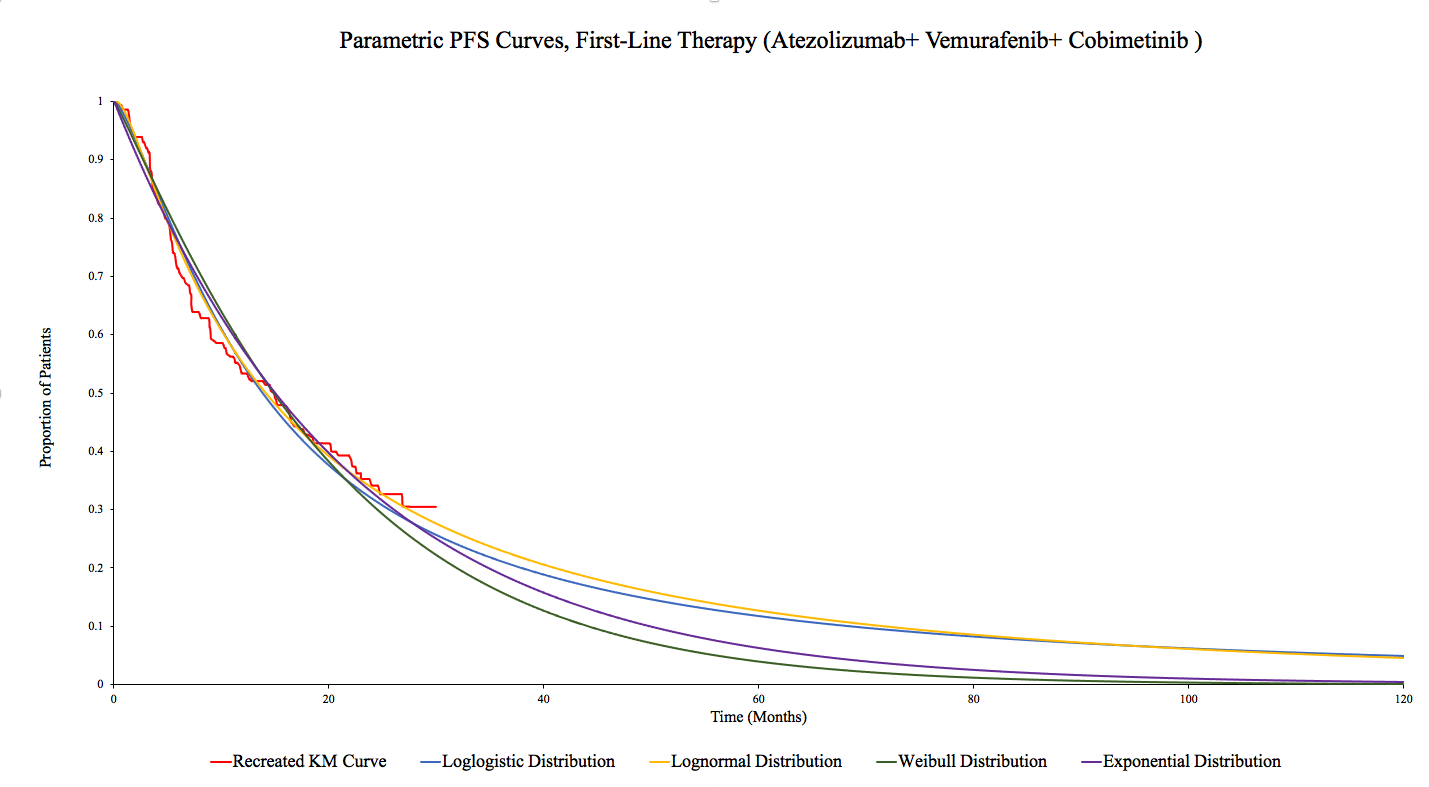


Supplementary figure 5: Parametric Distributions for First-Line Treatment (Vemurafenib + cobimetinib strategy). KM = Kaplan-Meier.


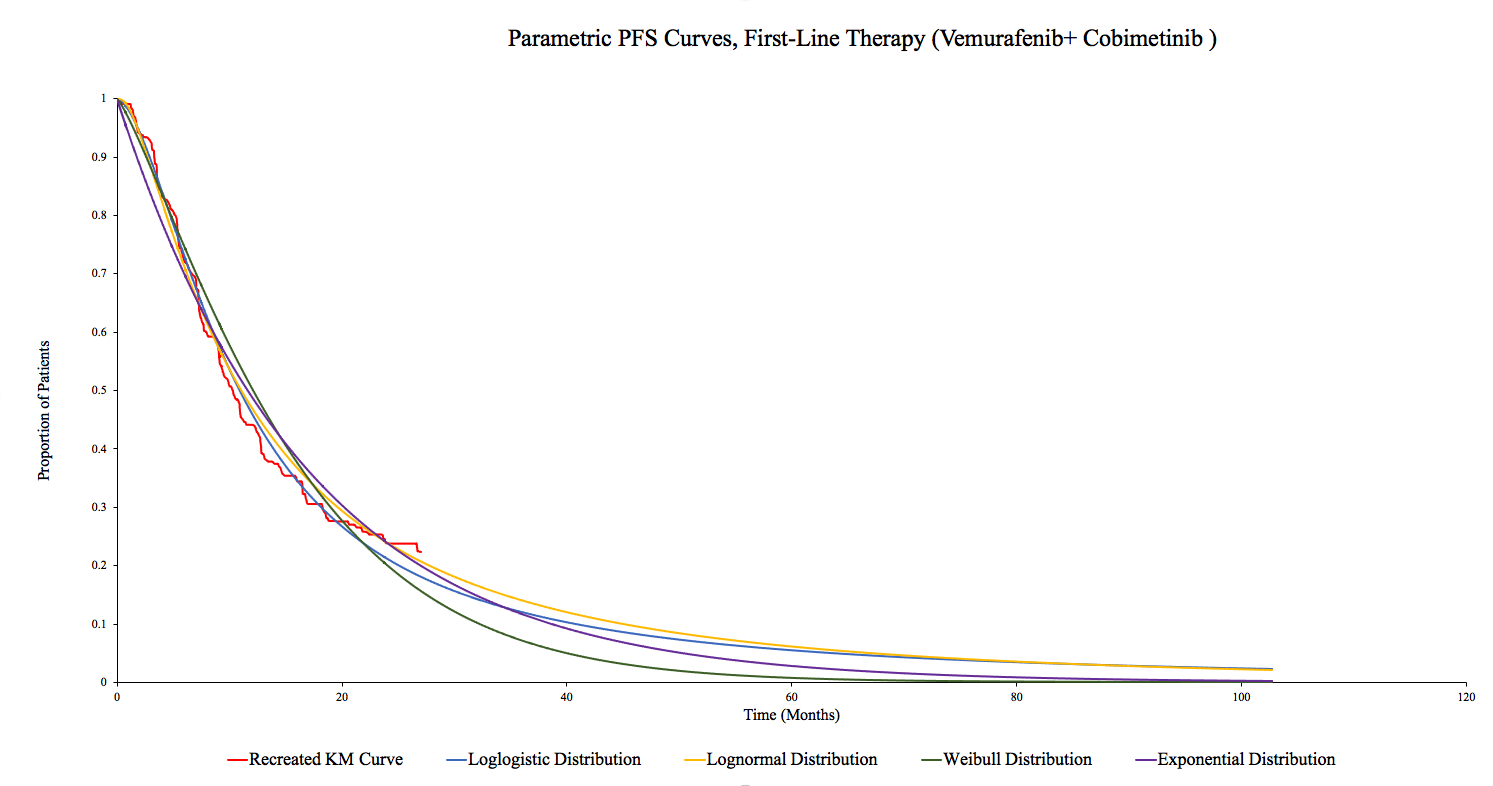


Supplementary figure 6: Parametric Distributions for First-Line Treatment (Dabrafenib + trametinib strategy). KM = Kaplan-Meier.


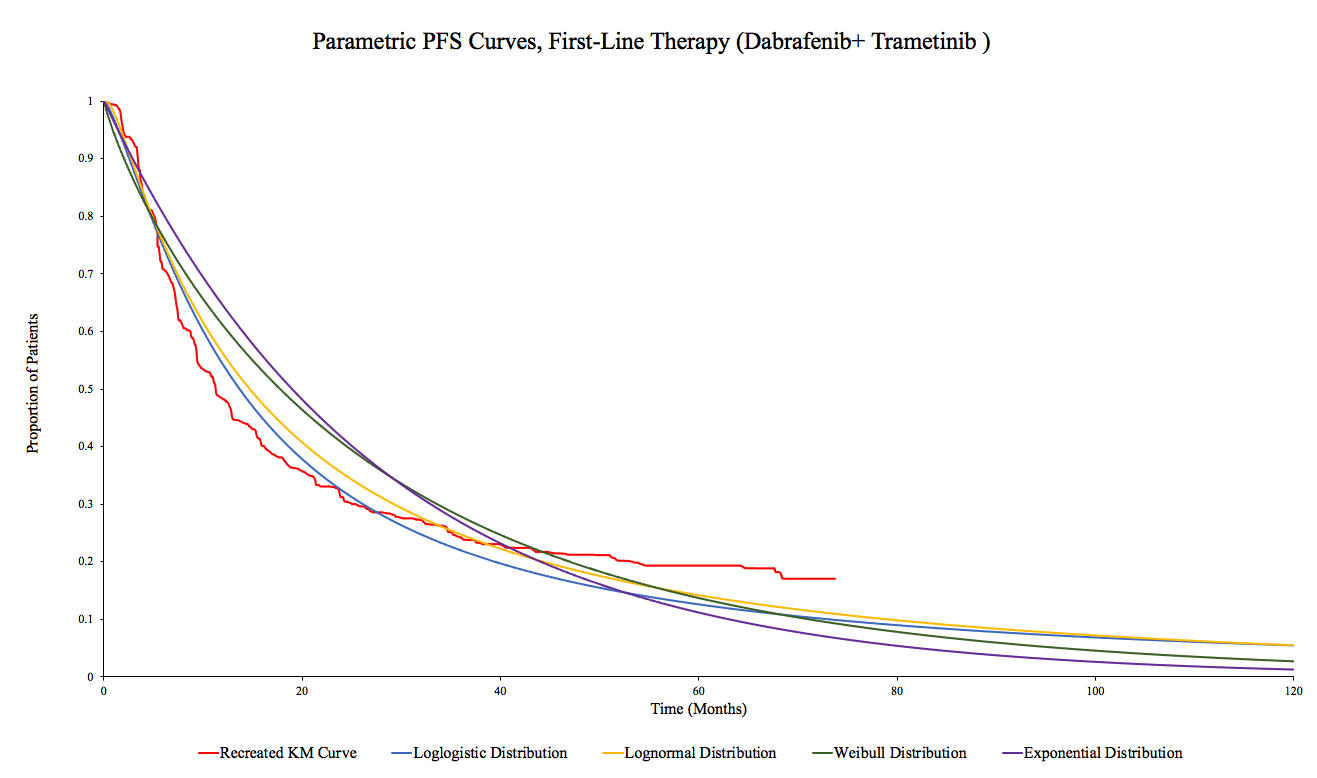


Supplementary figure 7: Parametric Distributions for First-Line Treatment (Encorafenib + binimetinib strategy). KM = Kaplan-Meier.


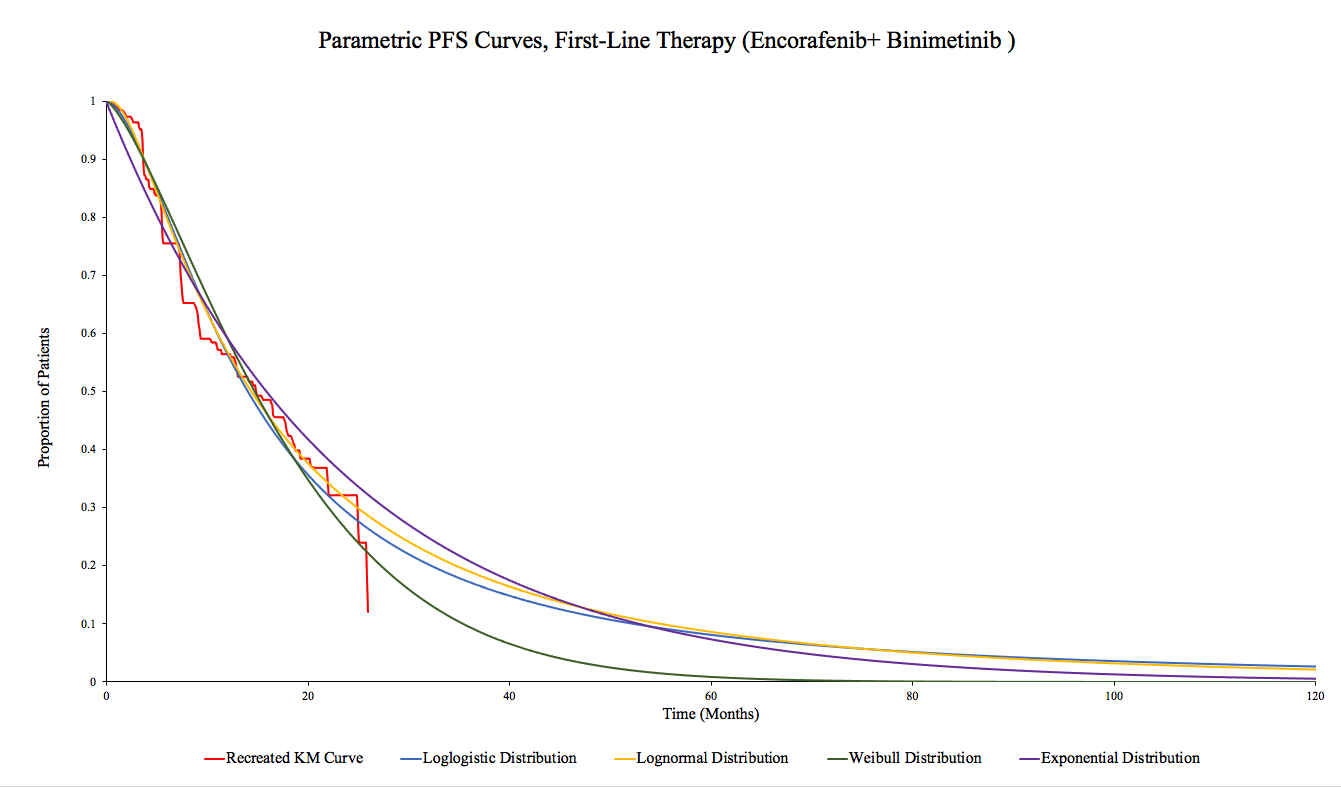


Supplementary figure 8: Parametric Distributions for Second-Line Treatment. KM = Kaplan-Meier.


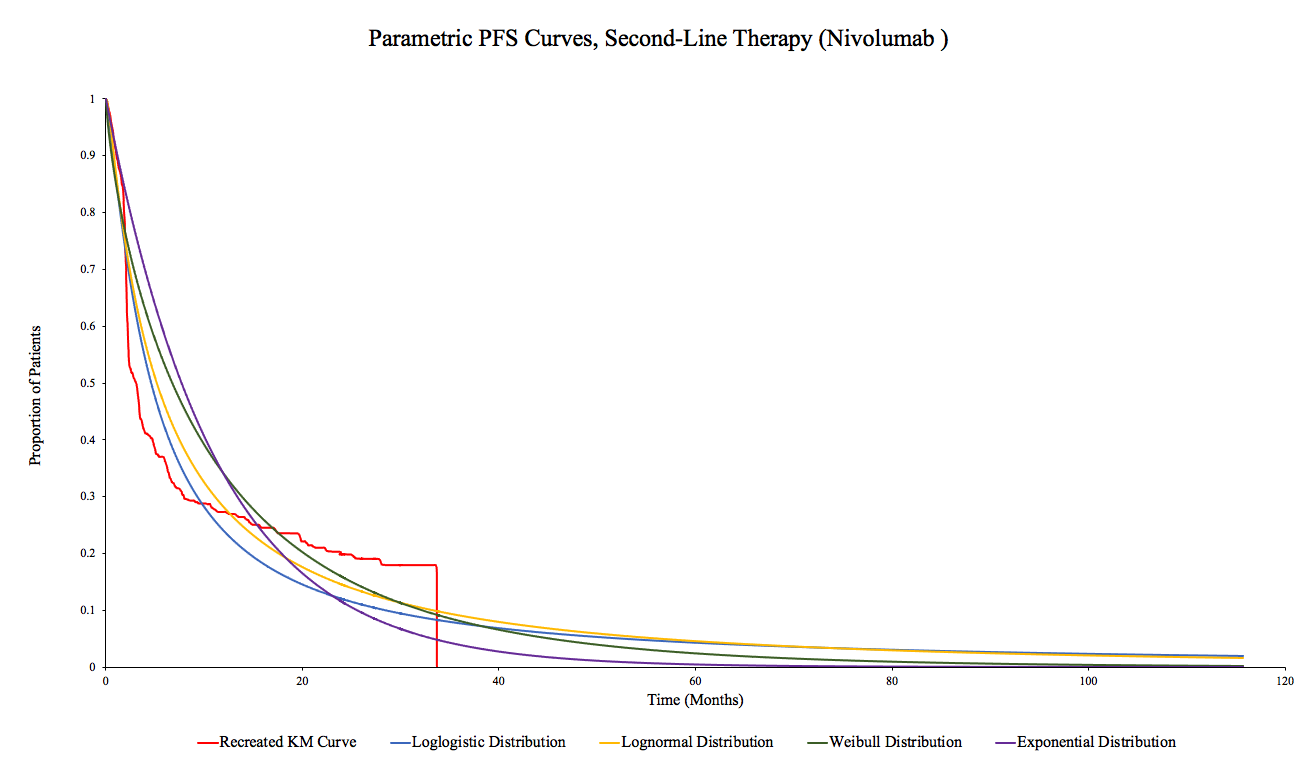


Supplementary figure 9: Parametric Distributions for Best Support Care State.

*BSC = Best support care.


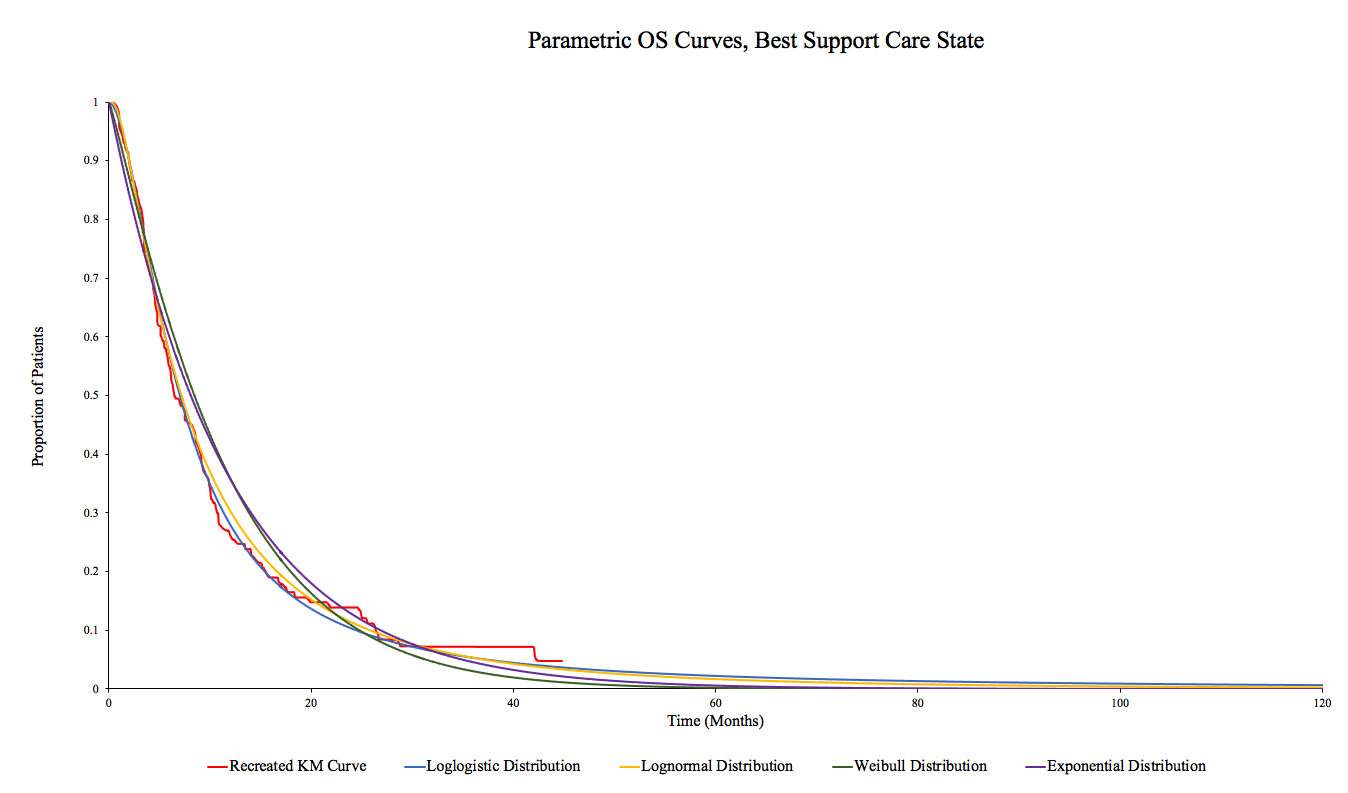


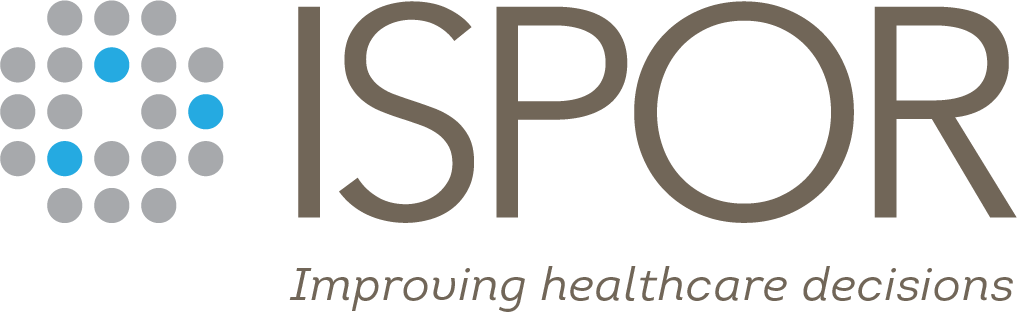

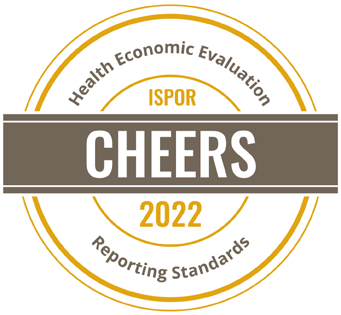


Consolidated Health Economic Evaluation Reporting Standards (CHEERS) 2022 Checklist

The CHEERS 2022 statement replaces the 2013 CHEERS statement, which should no longer be used. The CHEERS 2022 checklist contains 28 items with accompanying descriptions. Checklist users should indicate the section of the manuscript where relevant information can be found. The authors recommend using a section heading with a paragraph number. If an item does not apply to a particular economic evaluation, checklist users are encouraged to report “Not Applicable.” If information is otherwise not reported, checklist users are encouraged to write, “Not Reported.” Users should avoid the term “Not Conducted” as CHEERS is intended to guide and capture reporting. Additional information on CHEERS 2022 can be found [here.](https://www.ispor.org/heor-resources/good-practices/cheers)

# Title

## Title

Identify the study as an economic evaluation and specify the interventions being compared.

Page 1 (title page)

# Abstract

## Abstract

Provide a structured summary that highlights context, key methods, results, and alternative analyses.

Pages 2-3, lines 1-27.

# Introduction

## Introduction: Background and Objectives

Give the context for the study, the study question, and its practical relevance for decision making in policy or practice.

Background: pages 3-4, lines 28-60; objectives: page 4, lines 61-63.

# Methods

## Health economic analysis plan

Indicate whether a health economic analysis plan was developed and where available.

Pages 4-5, lines 65-82.

## Study population

Describe characteristics of the study population (such as age range, demographics, socioeconomic, or clinical characteristics).

Pages 5-6, lines 82-93.

## Setting and location

Provide relevant contextual information that may influence findings.

Page 4, lines 58-63

## Comparators

Describe the interventions or strategies being compared and why chosen.

Page 6, lines 71-76

## Perspective

State the perspective(s) adopted by the study and why chosen.

Page 4, line 63

## Time horizon

State the time horizon for the study and why appropriate.

Page 5, lines 66-68

## Discount rate

Report the discount rate(s) and reason chosen.

Page 5, line 80

## Selection of outcomes

Describe what outcomes were used as the measure(s) of benefit(s) and harm(s).

Page 5, lines 77-78

## Measurement of outcomes

Describe how outcomes used to capture benefit(s) and harm(s) were measured.

Page 5, lines 77-78

## Valuation of outcomes

Describe the population and methods used to measure and value outcomes.

Page 7, lines 113-130

## Measurement and valuation of resources and costs

Describe how costs were valued.

Page 8, lines 132-151

## Currency, price date, and conversion

Page 8, lines 132-151

1. **Rationale and description of model**

If modeling is used, describe in detail and why used. Report if the model is publicly available and where it can be accessed.

Pages 12-13, lines 233-236

## Analytics and assumptions

Describe any methods for analyzing or statistically transforming data, any extrapolation methods, and approaches for validating any model used.

Page 9, lines 154-169

## Characterizing heterogeneity

Describe any methods used for estimating how the results of the study vary for subgroups.

Page 9, lines 154-169

## Characterizing distributional effects

Describe how impacts are distributed across different individuals or adjustments made to reflect priority populations.

Page 9, lines 161-162

## Characterizing uncertainty

Describe methods to characterize any sources of uncertainty in the analysis.

Page 9, lines 154-169

## Approach to engagement with patients and others affected by the study

Describe any approaches to engage patients or service recipients, the general public, communities, or stakeholders (eg, clinicians or payers) in the design of the study.

Not applicable

# Results

## Study parameters

Report all analytic inputs (eg, values, ranges, references) including uncertainty or distributional assumptions.

Pages 9-11, lines 170-208

## Summary of main results

Report the mean values for the main categories of costs and outcomes of interest and summarize them in the most appropriate overall measure.

Pages 9-11, lines 170-208

## Effect of uncertainty

Describe how uncertainty about analytic judgments, inputs, or projections affects findings. Report the effect of choice of discount rate and time horizon, if applicable.

Pages 9-11, lines 170-208

## Effect of engagement with patients and others affected by the study

Report on any difference patient/service recipient, general public, community, or stakeholder involvement made to the approach or findings of the study.

Not applicable

# Discussion

## Study findings, limitations, generalizability, and current knowledge

Report key findings, limitations, ethical, or equity considerations not captured and how these could impact patients, policy, or practice.

Pages 11-13, lines 209-255

**Other Relevant Information**

1. **Source of funding**

Describe how the study was funded and any role of the funder in the identification, design, conduct, and reporting of the analysis.

Page 14, lines 276-278

1. **Conflicts of interest**

Report authors’ conflicts of interest according to journal or International Committee of Medical Journal Editors requirements.

Page 14, line 275

1. Gutzmer R, Stroyakovskiy D, Gogas H, et al. Atezolizumab, vemurafenib, and cobimetinib as first-line treatment for unresectable advanced BRAF(V600) mutation-positive melanoma (IMspire150): primary analysis of the randomised, double-blind, placebo-controlled, phase 3 trial. *Lancet (London, England).* 2020;395(10240):1835-1844.

2. Long GV, Hauschild A, Santinami M, et al. Adjuvant Dabrafenib plus Trametinib in Stage III BRAF-Mutated Melanoma. *N Engl J Med.* 2017;377(19):1813-1823.

3. Dummer R, Ascierto PA, Gogas HJ, et al. Overall survival in patients with BRAF-mutant melanoma receiving encorafenib plus binimetinib versus vemurafenib or encorafenib (COLUMBUS): a multicentre, open-label, randomised, phase 3 trial. *Lancet Oncol.* 2018;19(10):1315-1327.

4. Weber JS, D'Angelo SP, Minor D, et al. Nivolumab versus chemotherapy in patients with advanced melanoma who progressed after anti-CTLA-4 treatment (CheckMate 037): a randomised, controlled, open-label, phase 3 trial. *Lancet Oncol.* 2015;16(4):375-384.
